# Binary *Bacillus subtilis* protects the intestinal mucosa barrier and alleviates nonalcoholic steatohepatitis

**DOI:** 10.1002/ame2.12337

**Published:** 2023-07-20

**Authors:** Donglin Liu, Pengguo Chen

**Affiliations:** ^1^ Department of Gastroenterology Jiangxi Provincial People's Hospital, The First Affiliated Hospital of Nanchang Medical College Nanchang Jiangxi China

**Keywords:** non‐alcoholic steatohepatitis

## Abstract

**Background:**

Nonalcoholic steatohepatitis (NASH) is characterized by liver steatosis, inflammation, and even fibrosis. NASH is likely to develop into cirrhosis and liver cancer, the major causes of liver related deaths. We aimed to study the effect of probiotics on NASH via the gut‐liver axis.

**Methods:**

Thirty male Sprague–Dawley rats were divided into three groups. A control group of 10 rats was fed on a standard chow for 16 weeks. Twenty rats fed on a high‐fat diet for 8 weeks were separated to two groups: a model group (10 rats) fed on vehicle for 8 weeks and a treatment group (10 rats) supplemented with binary *Bacillus subtilis* for 8 weeks. Hepatic expression of IL‐6 and TNF‐ɑ and ileum expression of IL‐17 and occludin were measured.

**Results:**

The high‐fat diet caused inflammation of the liver and ileum in rats. Binary *Bacillus subtilis* treatment reduces liver inflammation through the intestinal liver axis. Increased levels of IL‐6 and TNF‐α were detected in rats fed a high‐fat diet, which were reduced to lower levels after treatment with binary *Bacillus subtilis*. In rats on the high‐fat diet, elevated IL‐17 levels and decreased occludin levels were observed. Treatment with *Bacillus subtilis* reduced IL‐17 levels and restored the expression of occludin.

**Conclusion:**

Binary *Bacillus subtilis* has a beneficial effect on liver inflammation and intestinal damage.

## Graphical abstract

The gut liver axis has a functional connection between the gastrointestinal tract and the liver. Most of the blood in the liver comes from the intestines. Binary Bacillus subtilis is composed of 4.5 × 10^8^ Fecal Escherichia coli R0026 and 5 × 10^7^ Bacillus subtilis R0179. Binary Bacillus Subtilis treatment protect the intestinal mucosa barrier and alleviate enteritis and non‐alcoholic steatohepatitis.

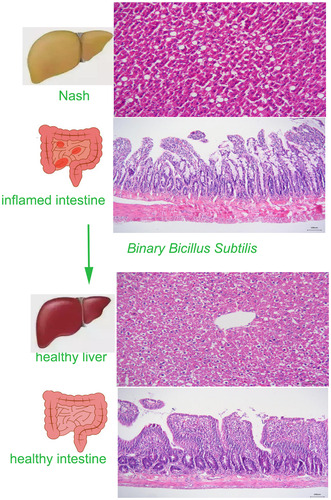

## 1 INTRODUCTION

Nonalcoholic fatty liver disease is a manifestation of metabolic syndrome and its global prevalence rate exceeds 25%.^1^ Nonalcoholic steatohepatitis (NASH) is a progressive form of lever disease characterized by steatosis, liver inflammation and fibrosis. NASH may eventually develop into cirrhosis and liver cancer, which are the main causes of liver related death.^2^, ^3^

A large number of studies exploring the pathogenesis and therapeutic targets of NASH have emerged, but there are currently no ideal therapeutic drugs or suitable therapeutic targets. In this study, we investigated the role of probiotics in the treatment of NASH. There is a reciprocal relationship between the liver and the intestine. Nutrients from intestinal sources are transported to the liver through the portal vein. In the liver, these substances interact with liver cells and immune cells. The liver is an immune organ, recruiting and activating immune cells to respond to various metabolic substances or pathogens. The interaction between the microbiome and the liver is crucial in the treatment of NASH.^4^

Intestinal microbiota mediate the progress of NASH by regulating intestinal inflammation and restoring barrier permeability. The intestinal microbiota form a complex ecosystem composed of over 1000 different bacteria.^5^ The main genera are *Bacteroides*, *Proteobacteria*, *Fusobacteria*, *Actinomycetes*, *Verrucaceae*, and *Cyanophyta*.^6^

Probiotics are living microorganisms such as *Lactobacillus*, *Bifidobacterium* and *Streptococcus* that produce health benefits. Many studies have shown that probiotics can improve the histological profile of NASH. *Bacillus subtilis* is a specialized aerobic bacterium, and previous studies have shown that binary *Bacillus subtilis* can improve inflammatory bowel disease.^7^, ^8^, ^9^, ^10^ In this study, we investigated the effect of *Bacillus subtilis* on NASH.

## 2 METHODS

### 2.1 Animal model and diets

Thirty male SD rats at 6 weeks of age were purchased. The rats were divided into three groups. A control group of 10 rats was fed on a standard chow for 16 weeks. The remaining 20 rats were fed on high‐fat diet for 8 weeks and were separated to two groups: a model group of 10 rats was fed on vehicle for 8 weeks, while a treatment group of 10 rats was administered with binary *Bacillus subtilis* (*E. faecium* R0026 4.5 × 10^8^ and *Bacillus subtilis* R0179 5 × 10^7^ per 250 mg body weight) (10 mg/kg/day) for 8 weeks.

## 3 REAGENTS AND ANTIBODIES

Antibodies against occludin and β‐actin and horseradish peroxidase‐conjugated anti‐mouse, anti‐rabbit anti‐rat, and anti‐goat secondary antibodies were purchased from Santa Cruz Biotechnology.

### 3.1 Hematoxylin and eosin and immunohistochemistry staining

The liver and ileum were fixed overnight in 10% phosphate buffered formalin acetate at 4°C. Paraffin sections were cut and stained with hematoxylin and eosin. Immunohistochemistry of liver and ileum sections was performed as previously described. Ileum sections were incubated with antibodies against IL‐17 and occludin. This procedure is performed as described previously.^11^, ^12^

### 3.2 Reverse transcription quantitative real‐time polymerase chain reaction

Total RNA was purified from liver using Trizol (Sigma) and reverse transcribed using PrimeScript RT Master Mix.(Takara). Reverse transcription quantitative PCR (RT‐qPCR) was conducted. Primer sequences were as follows: IL‐6 (forward: 5′‐CCAACTTCCAATGCTCTCCT‐3′; reverse: 5′‐ GTTTGCCGAGTAGACCTCA‐3′); TNF‐ɑ (forward: 5′‐CTCAAGCCCTGGTATGAGCC‐3′; reverse: 5′‐GGCTGGGTAGAGAACGGATG‐3′); β‐actin (forward: 5′‐GCCATGTACGTAGCCATCCA‐3′; reverse: 5′‐GAACCGCTCATTGCCGATAG‐3′). Gene expression was measured by the 2^−ΔΔCT^ method.^12^

### 3.3 Detection of ALT and AST

Serum levels of aminotransferase aspartate (ALT) and aminotransferase (AST) in rats were measured using ELISA kits (Jiancheng Bioengineering, CN) according to the manufacturer's protocol.

### 3.4 Immunoblots

Immunoblotting analysis was carried out as described previously. Immunoblots were visualized with a chemiluminescence detection kit.^12^

### 3.5 Statistical analysis

The data are expressed as the means ± SEM. Statistical significance was evaluated by one‐way ANOVA. Differences were considered significant at a *p* value < 0.05.

## 4 RESULTS

### 4.1 Binary *Bacillus subtilis* attenuates hepatic steatosis in rats fed a high‐fat diet

**FIGURE 1:**
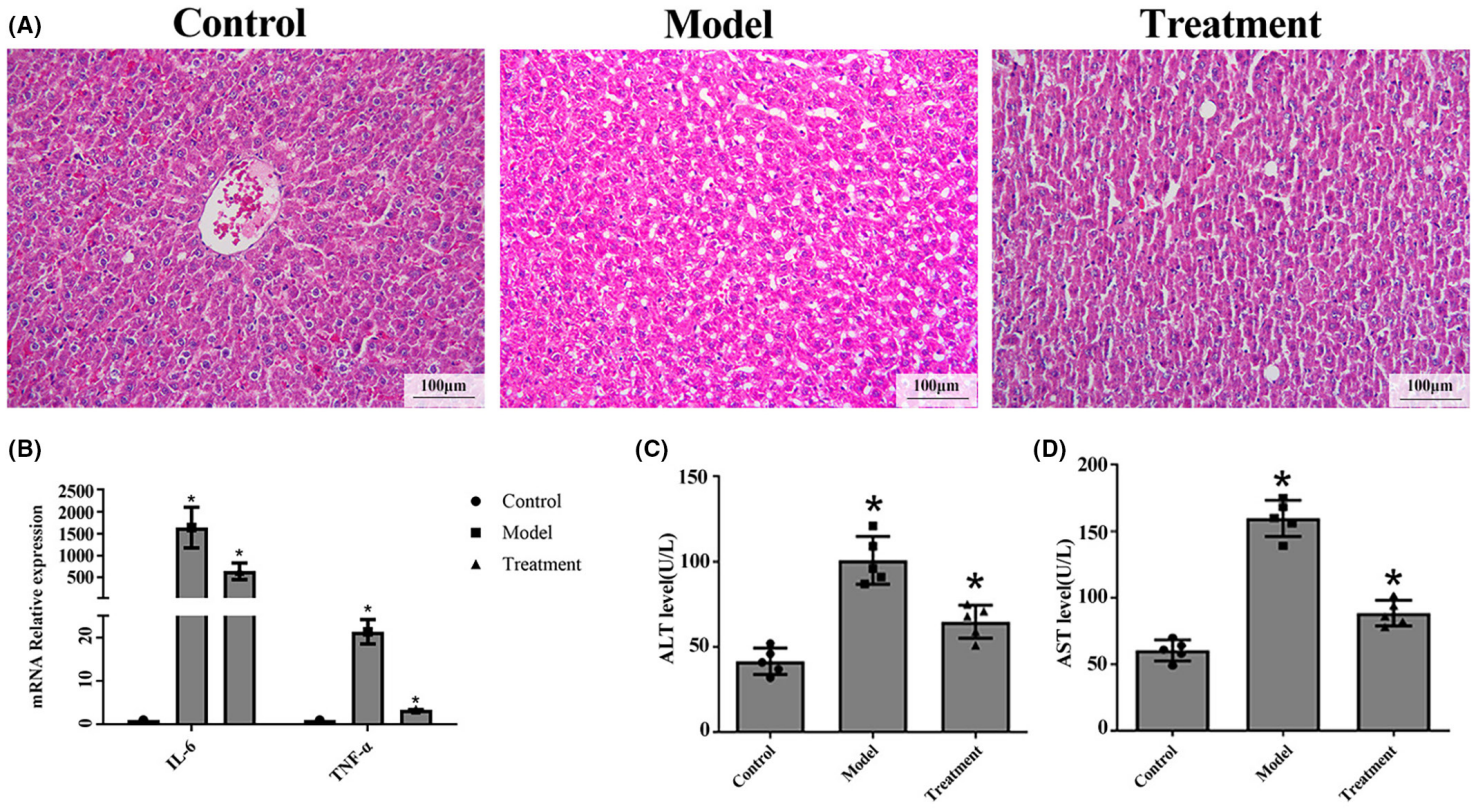
Administration of binary *Bacillus subtilis* protects against hepatic steatosis in rats fed a high‐fat diet. (A) Representative pictures of the livers and hematoxylin and eosin staining of the liver sections are shown (scale bars, 100 μm). (B) The expression of IL‐6 and TNF‐ɑ by RT‐PCR analysis. (C) and (D) ALT (C) and AST (D) levels in rat sera from each group collected at the end of the study.

We explored the effect of binary *Bacillus subtilis* on NASH. As shown in Figure 1A, feeding rats a high‐fat diet resulted into liver inflammation with hepatic enlargement and discoloration. The inflammation cytokines IL‐6 and TNF‐ɑ mRNA levels increased compared to the rats in control group. After binary *Bacillus subtilis* treatment liver inflammation was alleviated and IL‐6 and TNF‐ɑ mRNA levels decreased, which indicated a beneficial effect of binary *Bacillus subtilis* in alleviating steatohepatitis (Figure 1B). The levels of ALT and AST in plasma were upregulated in the high‐fat diet rats, and binary *Bacillus subtilis* attenuated expression of these biomarkers (Figure 1C,D).

### 4.2 Ileal inflammation is reduced by administration of binary *Bacillus subtilis*

**FIGURE 2:**
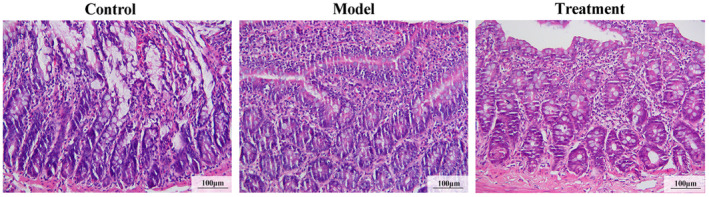
Binary *Bacillus subtilis* improved intestinal epithelial barrier damage in rats fed a high‐fat diet. Representative pictures of hematoxylin and eosin staining of the ileal sections from each group of rats are shown (scale bars, 100 μm).

We further studied the association between liver inflammation and ileal damage. In high‐fat‐fed rats, ileitis was associated with steatohepatitis. As shown in Figure 2, ileal inflammation with loss of ileac epithelium villus structure was observed, and the ileal epithelium damage was reversed after binary *Bacillus subtilis* treatment (Figure 2). This implies that binary *Bacillus subtilis* attenuates liver inflammation by reversing mucosal lesions.

### 4.3 Occludin expression was increased after administration of binary *Bacillus subtilis* in rats fed a high‐fat diet

**FIGURE 3:**
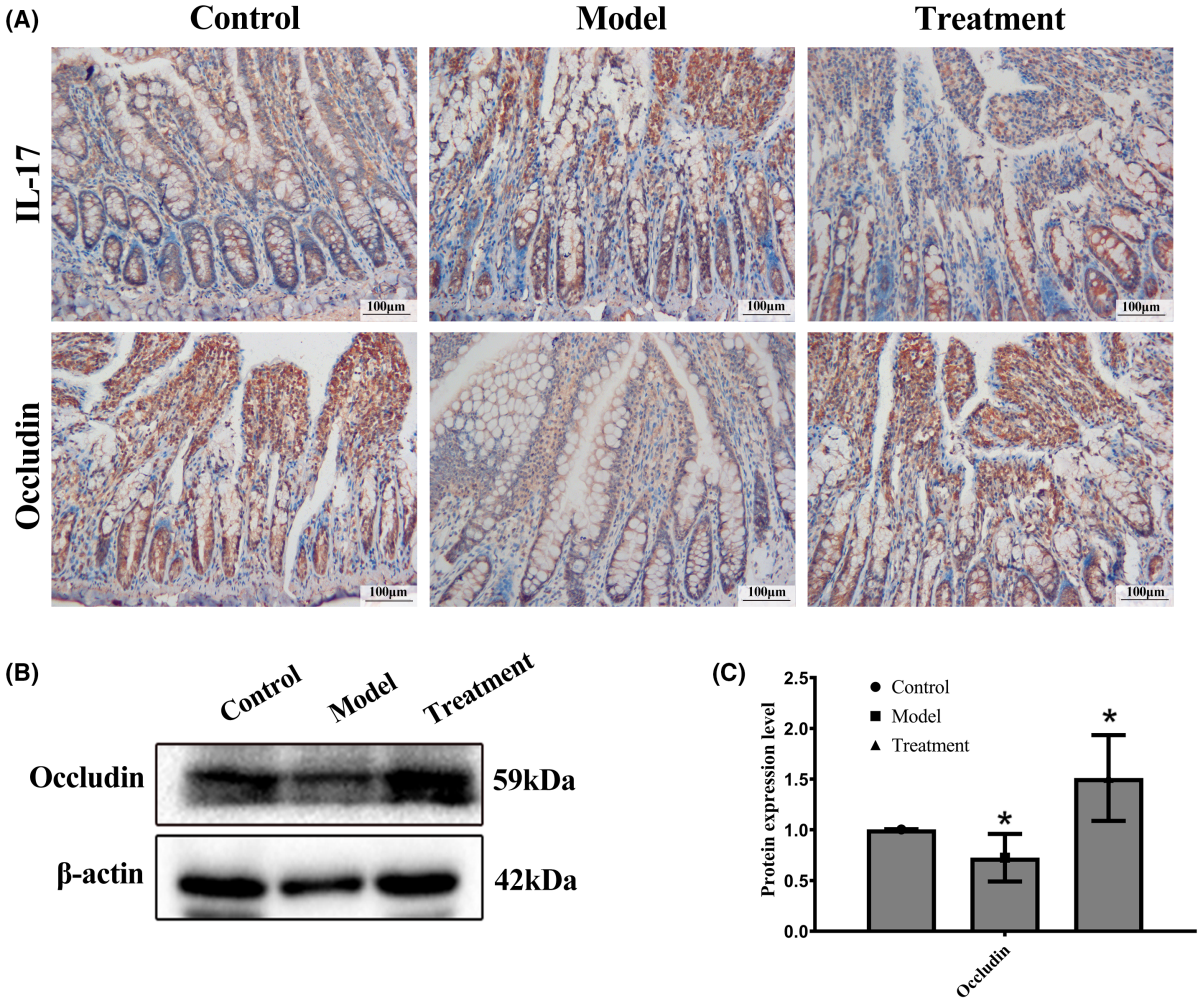
Damage to the intestinal epithelial barrier is reversed by administration of binary *Bacillus subtilis*. (A) Representative immunostaining of zonula IL‐17 and occludin in the ilea is shown. (B) Immunoreactive bands of occludin. (C) Relative density of occludin.

Ileal tight junctions are located on the outer side of the ileum and close the intercellular gap between adjacent cells. IL‐17 is an inflammatory factor,^13^ while occludin is expressed in epithelial cells and has regulatory role on tight junctions and signaling transduction.^14^, ^15^, ^16^ A high‐fat diet results in ileal damage. In our study, IL‐17 expression increased and occludin expression decreased in high‐fat‐fed rats, as demonstrated by immunostaining and Western blots. After binary *Bacillus subtilis* treatment IL‐17 levels decreased and occludin levels recovered. These results showed that binary *Bacillus subtilis* reversed ileal damage and reduced the inflammation. (Figure 3A,B).

## 5 DISCUSSION

Most blood in the liver originate from the intestines. The gut‐liver axis forms a functional connection between the digestive tract and the liver. Damage to this axis leads to the pathogenesis of NASH.^17^ Changes in the microbiota balance gut homeostasis and regulate host metabolism. Changes in intestinal microbiota and enhanced bacterial endotoxin translocation are common in the development of NASH. Kupffer cells, neutrophils, hepatocytes and stellate cells are activated and then release large amounts of inflammatory mediators like TNF‐α and IL‐6.^18^

Binary *Bacillus subtilis* is composed of 4.5 × 10^8^ fecal *Escherichia coli* R0026 and 5 × 10^7^ *Bacillus subtilis* R0179. Our research shows that *Bacillus subtilis* binary reduces liver inflammation caused by a high‐fat diet, by reducing the proinflammatory cytokines IL‐6 and TNF‐α Expression in liver tissue. The intestinal epithelium prevents the microbiota and its products from entering the bloodstream. In high‐fat‐fed rats, intestinal epithelial damage leads to translocation of intestinal microbial products, resulting in NASH.^19^, ^20^, ^21^ In our study, rats fed with high fat experienced intestinal injury and increased expression of the proinflammatory cytokine IL‐17. After treatment with *Bacillus subtilis* binary, compared with model rats fed high‐fat, intestinal injury was reversed and IL‐17 levels increased.

The integrity of the intestinal mucosa depends on two components: the protective layer of the intestinal epithelium and the tight connection between intestinal epithelial cells and intestinal immune cells. The tight junctions (TJs) seal intestinal epithelial cells and can prevent the exit of intestinal microbiota and its metabolites such as endotoxin and lipopolysaccharide (LPS). Tight junctions include proteins such as occludin. The loss of tight junction proteins leads to intestinal permeability.^16^, ^22^, ^23^ Our data suggest that when the gut is damaged, the expression of occludin decreases. After treatment with *Bacillus subtilis*, the ileal injury was reversed and the expression of occludin increased.

The use of probiotics is a promising strategy for regulating the intestinal microbiota and has beneficial effects for NASH. This study demonstrates the effectiveness of probiotics in NASH. Microflora modulators can play an important adjuvant therapeutic role in pathological processes involving NASH by improving the intestinal barrier.

## AUTHOR CONTRIBUTIONS

Donglin liu participated in animal and molecular experiments, Pengguo Chen is involved with molecular experiments, data analysis and article writing.

## 6 ACKNOWLEDGMENTS

We are thankful for the funding support from the Natural Science Foundation of Jiangxi Province (20171BAB205011, 20202BABL206014).

## CONFLICT OF INTEREST STATEMENT

All authors are in agreement with the content of the manuscript. There is no conflict of interest.

## EHICS STATEMENT

All animal experiment protocols were approved by the Animal Care and Use Committee of Jiangxi Provincial People’s Hospital (2020091).
